# *UQCRC1* variants in early-onset and familial Parkinson's disease in a Taiwanese cohort

**DOI:** 10.3389/fneur.2022.1090406

**Published:** 2022-12-09

**Authors:** Ting-Wei Liao, Chih-Ying Chao, Yih-Ru Wu

**Affiliations:** ^1^Department of Neurology, Linkou Chang Gung Memorial Hospital, Taoyuan, Taiwan; ^2^Department of Neurology, College of Medicine, Chang Gung University, Taoyuan, Taiwan

**Keywords:** ubiquinol-cytochrome C reductase core protein 1 (*UQCRC1*), Parkinson's disease, early-onset, familial, parkinsonism

## Abstract

**Background:**

A recent Taiwanese study reported variants of the ubiquinol-cytochrome c reductase core protein 1 (*UQCRC1*) gene linked to autosomal dominant parkinsonism with polyneuropathy. This study investigated the pathogenicity of *UQCRC1* in a Taiwanese cohort of patients with Parkinson's disease (PD).

**Method:**

This study involved 107 participants (98 with early-onset PD and nine with familial PD). All *UQCRC1* coding exons and exon–intron boundaries were sequenced. The rarity and pathogenicity of the identified variants were analyzed. The carrier frequencies of our cohort and the Taiwan Biobank were compared through a Pearson's χ^2^ or Fisher's exact test along with Bonferroni corrections.

**Results:**

Three missense variants (c.643G > C, p.D215H; c.800C > G, p.P267R, and c.923A > G, p.N308S) and seven rare variants were identified. No significant differences in the missense-variant carrier frequency were noted between our cohort and individuals in the Taiwan Biobank. Furthermore, no significant associations were noted between the variants and the risk of PD.

**Conclusions:**

Our study is not supporting a role of *UQCRC1* variants in PD.

## Introduction

Parkinson's disease (PD) is the second most common neurodegenerative disorder and is caused by an interaction of aging with environmental and genetic factors. The genetics of PD have been extensively studied over the past 20 years. However, the common monogenetic PD accounts for only 30% of familial and 3–5% of sporadic cases (1). One recent meta-analysis of genome-wide association studies (GWASs) on PD revealed 90 notable independent genome-wide risk variants (2). These variants only contribute to 16–36% of the heritable risk of PD. Considerable risk of pathogenic variants associated with the progression of PD has yet to be discovered.

Recently, Lin et al. reported that the variants of the ubiquinol-cytochrome c reductase core protein 1 (*UQCRC1*) gene were linked to autosomal dominant parkinsonism with polyneuropathy (3, 4). After the c.941A > C (p.Y314S) variant was identified in an index family (3), c.931A > C (p.I311L) and the allele with concomitant c.70-1G4A and c.73_73insG (p.A25Gfs^*^2) variants were further identified in two other families with PD in this study. The affected individuals presented with asymmetrical tremor predominant parkinsonism and had a mean onset age of 57.5 years (range: 42–69 years). Images from Tc-99m TRODAT single-photon emission computed tomography indicated asymmetric loss of the dopamine transporter. Nerve conduction studies revealed axonal-type predominant sensorimotor polyneuropathy. All patients exhibited satisfactory responses to levodopa (4).

Mitochondrial dysfunction is a key in the pathogenesis of idiopathic and monogenetic PD (5). PD-associated genes, such as *PRKN, PINK1*, and *DJ-1*, are directly involved in mitochondrial functions. Although *LRRK2, SNCA*, and *GBA* indirectly affect the electron transport chain through the regulation of lysosomal functions, lipid metabolism, and protein aggregation (6). *UQCRC1* is a core subunit of the mitochondrial respiratory chain complex III (7). *UQCRC1* p.Y314S may disrupt the activity of mitochondrial respiratory chain complex III in the neurons and may be associated with impaired locomotion, dopaminergic neuronal loss, and peripheral nerve degeneration (4). The knockout of *UQCRC1* also affects brain ischemic tolerance and is involved in apoptotic neurodegeneration (8, 9).

As *UQCRC1* p.Y314S was first reported among a Taiwanese family, this study investigated the pathogenicity of *UQCRC1* variants in a Taiwanese cohort of patients with early-onset or familial PD.

## Materials and methods

### Patients

This study involved 107 participants, including 98 patients with early-onset PD (EOPD, defined by an age at onset of ≤ 50 years) and 9 patients with familial PD (FPD, defined as those having at least one first-degree or second-degree relative who has PD). The mean age at onset among patients with EOPD was 43.78 ± 5.67 years (range: 27–50 years), and the mean age at onset among patients with FPD was 55.00 ± 9.49 years (range: 39–65 years). All patients were examined by an experienced movement disorder specialist (YR Wu) and diagnosed according to the UK Parkinson's Disease Society Brain Bank clinical diagnostic criteria (10).

The cohort in this study did not overlap with the Taiwanese cohorts in Lin's et al. (3, 4). The study was approved by the Institutional Review Board of Chang Gung Memorial Hospital (Ethical License No. 201701458B0C601). Written informed consent was obtained from all participants.

### Genetic analysis

DNA was isolated from the leucocytes of the participants per standard protocols. Large deletions or duplications of common PD-related genes including *SNCA, PRKN, PINK1, DJ-1, ATP13A2, PLA2G6, FBXO7*, and *LRRK2* were detected using the multiplex ligation-dependent probe amplification. Participants, except four, received either whole-genome sequencing or designed next-generation sequencing panel, containing 51 genes associated with parkinsonism (Supplementary Table 1). Patients were excluded if they had pathogenic variants of these genes. We sequenced all the *UQCRC1*-coding exons and exon–intron boundaries using the polymerase chain reaction primers listed in Supplementary Table 2. We examined the rarity and pathogenicity of the identified variants. Rarity was indicated by a minor allele frequency (MAF) of <0.1% according to the allele frequency in the 1,000 Genomes, the Genome Aggregation Database (GnomAD), and the Taiwan Biobank database (11–13). Pathogenicity was predicted using online *in silico* tools. Pathogenicity classification was evaluated according to the standards and guidelines of the American College of Medical Genetics and Genomics (ACMG) using an online version of InterVar and VarSome (14–16).

### Statistics

We used a Pearson's χ^2^ test or Fisher's exact test to compare the frequencies of carriers between our participants and the enrolees of the Taiwan Biobank database. Because this study tested 12 variants, we applied the Bonferroni correction for multiple comparisons. Two-tailed *p* < 0.00417 were considered statistically significant. Statistical analysis was performed using IBM SPSS Statistics (version 26.0., IBM, Armonk, NY, USA).

## Results

Supplementary Table 3 summarizes the *UQCRC1* variants identified in our study. No previously reported potentially pathogenic variants were identified in our patients. Three missense variants (c.643G > C, p.D215H; c.800C > G, p.P267R, and c.923A > G, p.N308S) were identified (Table 1). Two of those variants (p.D215H and p.P267R) exhibited a relatively high allele frequency (MAF > 3%) among East Asian and Taiwanese populations. Less than half the *in silico* tools indicated a pathogenic consequence in the three missense variants. The *in silico* pathogenicity predictions are detailed in Supplementary Table 4. Seven rare variants (with MAF <0.1% in all databases, if available) were identified, including four intron variants, two non-coding transcript variants, and one synonymous variant (Supplementary Tables 5, 6). No association of the variants with changes in the splice site change was indicated; in addition, the variants were not strongly conserved across species. Pathogenicity was classified according to the ACMG criteria, as displayed in Table 1, Supplementary Tables 5, 6.

**Table 1:** Missense variants of *UQCRC1* identified among 98 patients with EOPD and nine with FPD.

**Genomic location (GRCh38.p12)**	**dbSNP ID**	**Nucleotide change**	**AA change**	**1000G MAF (S. Han)**	**GnomAD MAF (East Asian)**	**Taiwan biobank MAF**	**Allele F in our study**	***p*-value**	Pathogenic functional predictions*(total)	**ACMG pathogenicity classification**
Chr3: 48,603,627	rs17080284	c.643G > C	p.Asp215His (p.D215H)	G = 0.0143	G = 0.03331	G = 0.036915	0.0280	0.573	6 (13)	Uncertain significance
Chr3: 48,601,374	rs149245457	c.800C > G	p.Pro267Arg (p.P267R)	C = 0.0381	C = 0.03985	C = 0.035620	0.0327	0.852	3 (13)	Uncertain significance
Chr3: 48,601,018	rs187641562	c.923A > G	p.Asn308Ser (p.N308S)	C = 0.0048	C = 0.003865	C = 0.004944	0.0047	1.000	4 (13)	Uncertain significance

* *In silico* prediction tools involved the use of FATHMM (functional analysis through hidden Markov models), Mutation Assessor, SIFT (sorting intolerant from tolerant), SIFT4G, MutationTaster, PolyPhen2-HDIV(polymorphism phenotyping v2 human diversity), PolyPhen2-HVAR (polymorphism phenotyping v2 human variation), PROVEAN (Protein Variation Effect Analyzer), CADD (combined annotation-dependent depletion), M-CAP (Mendelian clinically applicable pathogenicity), LRT (Likelihood Ratio Test), PrimateAI, and REVEL score.

EOPD, early-onset Parkinson's disease; FPD, familial Parkinson's disease; GRCh38.p12, Genome Reference Consortium Human Build 38 patch release 12; dbSNP, The Single Nucleotide Polymorphism Database; AA, Amino acid; MAF, minor allele frequency; 1000G, 1000 Genomes; S. Han, Southern Han Chinese; gnomAD, The Genome Aggregation Database; F, frequency; ACMG, American College of Medical Genetics and Genomics.

*p* < 0.00417 were considered statistically significant after Bonferroni's correction was applied.

Among the *UQCRC1* variants identified in this study, the allele frequencies of only 12 variants were reported in the Taiwan Biobank database. The prevalence of c.297 + 21C > T was higher among our participants than among the enrollees of the Taiwan Biobank database (*p* = 0.025), but this difference was non-significant after the Bonferroni correction was applied (Supplementary Table 3). The frequencies of carriers of the remaining 11 variants identified in both groups were similar.

## Discussion

In this study, we performed a comprehensive mutational screening of *UQCRC1* in a Taiwanese cohort of patients with EOPD and FPD. We identified no pathogenic variants potential associated with the risk of PD.

Although we identified three missense variants (c.643G > C, p.D215H; c.800C > G, p.P267R, and c.923A > G, p.N308S) among our participants, their pathogenicity was low, as indicated by the high allele frequency in East Asian populations and benign functional predictions. The missense variants were also recently reported in a cohort in eastern China, and the frequencies were similar between patients with PD and controls (17). Although the prevalence of c.297 + 21C > T was higher among our participants than among the enrolees of the Taiwan Biobank database, *in silico* pathogenicity predictors indicated that c.297 + 21C > T does not notably affect the mRNA splicing of *UQCRC1*. Allele frequencies more than 1% in East Asian populations are higher than expected for autosomal dominant disorders.

Recently, Senkevich et al. studied common and rare *UQCRC1* variants according to GWAS and whole-genome sequencing data obtained from two large European cohorts (18). No common or rare variants in *UQCRC1* were revealed to be associated with the risk of PD. Courtin et al. sampled 241 patients with FPD from 163 families with targeted or whole-exome sequencing; most of the patients were Europeans of European descent (19). They found no disease-causing variants of *UQCRC1* among the study participants. Zhao et al. recruited 477 patients with FPD, 1,440 patients with sporadic EOPD, and 1,357 ethnicity-matched controls in their study; all participants were recruited from mainland China (20). In the study, a rare damaging variant (c.330G > C, p.E110D) was identified in one patient with sporadic EOPD, and one common synonymous variant (c.1419C > T, p.S473=) was revealed to be significantly associated with PD susceptibility. Furthermore, rs182453765 (p.S473=) was identified among four patients with EOPD in the present study. These results should be interpreted with caution and further validated with multiethnic cohorts.

The present study has a few limitations. First, only 107 patients with EOPD or FPD were involved. The sample size was insufficient for detecting significant genetic variants among the groups. Second, the onset ages of *UQCRC1* heterozygotes carriers were reported to be 42–69 years by Lin et al. (4). Potential late-onset sporadic cases may have been excluded from our genetic study. Third, electrophysiologic data were not available for assessing polyneuropathy among the participants with *UQCRC1* variants.

Overall, the results of this study indicate that the *UQCRC1* gene may not be associated with EOPD or FPD among the Taiwanese population. Future studies with larger sample sizes and functional investigation are warranted to further understand the potential role of *UQCRC1* in the onset of PD.

## Data availability statement

The data presented in the study are deposited in the Figshare repository, doi: 10.6084/m9.figshare.21525783.

## Ethics statement

The studies involving human participants were reviewed and approved by the Institutional Review Board of Chang Gung Memorial Hospital (Ethical License No. 201701458B0C601). The patients/participants provided their written informed consent to participate in this study.

## Author contributions

Y-RW: conceptualization, methodology, investigation, resources, writing—review and editing, supervision, project administration, and funding acquisition. T-WL: software and writing—original draft preparation. C-YC: validation, formal analysis, and visualization. T-WL and Y-RW: data curation. All authors have read and agreed to the published version of the manuscript.
