# Supplementary material for: UQCRC1 variants in early-onset and familial Parkinson's disease in a Taiwanese cohort
Source: Front Neurol. 2022 Dec 9;13:1090406. doi: 10.3389/fneur.2022.1090406 (PMC9780373; doi:10.3389/fneur.2022.1090406)
Supplement: Supplementary file 1 [file Data_Sheet_1.docx]

| **Supplementary Table 1.** 51 parkinsonism-related genes covered by next generation sequencing panel | | | | |
| --- | --- | --- | --- | --- |
| *VPS35* | VPS35 retromer complex component |  | *TYROBP* | transmembrane immune signaling adaptor TYROBP |
| *GBA* | glucosylceramidase beta |  | *FBXO7* | F-box protein 7 |
| *PINK1* | PTEN induced kinase 1 |  | *VCP* | valosin containing protein |
| *GIGYF2* | GRB10 interacting GYF protein 2 |  | *MAPT* | microtubule associated protein tau |
| *LRRK2* | leucine rich repeat kinase 2 |  | *PTRHD1* | peptidyl-tRNA hydrolase domain containing 1 |
| *UCHL1* | ubiquitin C-terminal hydrolase L1 |  | *CHMP2B* | charged multivesicular body protein 2B |
| *PRKN* | parkin RBR E3 ubiquitin protein ligase |  | *ATP13A2* | ATPase cation transporting 13A2 |
| *SNCA* | synuclein alpha |  | *PARK7* | Parkinsonism associated deglycase |
| *CSMD1* | CUB and Sushi multiple domains 1 |  | *TARDBP* | TAR DNA binding protein |
| *DNAJC13* | DnaJ heat shock protein family (Hsp40) member C13 |  | *CHCHD2* | coiled-coil-helix-coiled-coil-helix domain containing 2 |
| *DCTN1* | dynactin subunit 1 |  | *RAB39B* | RAB39B, member RAS oncogene family |
| *PLA2G6* | phospholipase A2 group VI |  | *SYNJ1* | synaptojanin 1 |
| *POLG* | DNA polymerase gamma, catalytic subunit |  | *SOD1* | superoxide dismutase 1 |
| *VPS13C* | vacuolar protein sorting 13 homolog C |  | *COQ2* | coenzyme Q2, polyprenyltransferase |
| *PODXL* | podocalyxin like |  | *PANK2* | pantothenate kinase 2 |
| *TMEM230* | transmembrane protein 230 |  | *NPC2* | NPC intracellular cholesterol transporter 2 |
| *FMR1* | FMRP translational regulator 1 |  | *EIF4G1* | eukaryotic translation initiation factor 4 gamma 1 |
| *ADCY5* | adenylate cyclase 5 |  | *SMPD1* | sphingomyelin phosphodiesterase 1 |
| *ATP1A3* | ATPase Na+/K+ transporting subunit alpha 3 |  | *GRN* | granulin precursor |
| *TREM2* | triggering receptor expressed on myeloid cells 2 |  | *HTRA2* | HtrA serine peptidase 2 |
| *FUS* | FUS RNA binding protein |  | *ATN1* | atrophin 1 |
| *OPTN* | optineurin |  | *PRNP* | prion protein |
| *TAF1* | TATA-box binding protein associated factor 1 |  | *APP* | amyloid beta precursor protein |
| *DNAJC6* | DnaJ heat shock protein family (Hsp40) member C6 |  | *PSEN1* | presenilin 1 |
| *GCH1* | GTP cyclohydrolase 1 |  | *PSEN2* | presenilin 2 |
| *NPC1* | NPC intracellular cholesterol transporter 1 |  |  |  |

| **Supplementary Table 2.** PCR primers for *UQCRC1* genomic regions | | |
| --- | --- | --- |
| Primer name | Primer sequence 5' > 3' | Amplicon size (bp) |
| UQCRC1_PF | AGGCTGGTCTTGGACTCCTC | 1320 |
| UQCRC1_2R | CAAACCACAAACGGGAAGAC |  |
| UQCRC1_3F | CAGCTCTCCCTGACTCCAG | 398 |
| UQCRC1_3R | AGGGGCTCATCAGATACGTG |  |
| UQCRC1_4F | TGAGGGAGGTACCTGTGGAC | 843 |
| UQCRC1_5R | TTCAAGTGAAGCCCACAAAG |  |
| UQCRC1_6F | GAAGTCAGGCATGAGGCAAG | 499 |
| UQCRC1_6R | TGTAACCAGTCCCAGTGGTG |  |
| UQCRC1_7F | CTTTTGCTCTCCTGGGACTG | 824 |
| UQCRC1_8R | CTTCAACGCACACTGTGACC |  |
| UQCRC1_8F | ACACCCTCTACCCACACCTG | 956 |
| UQCRC1_10R | CTCCCTGACCTCATGCTGAC |  |
| UQCRC1_11F | TCAGGGAGGTCCTTGTCAAC | 794 |
| UQCRC1_12R | CTGGGACAGGTTTTCTGAGC |  |
| UQCRC1_13F | ACATGAAGGGACAGGCTCAG | 580 |
| UQCRC1_13R | CTCCCCTAAGTGGCTGAGTG |  |
| PCR, polymerase chain reaction | |  |

| **Supplementary Table 3.** Variants of the *UQCRC1* gene identified among 98 patients with EOPD and 9 with FPD. | | | | | | | | | | | |  |
| --- | --- | --- | --- | --- | --- | --- | --- | --- | --- | --- | --- | --- |
| **Genomic location (GRCh38.p12)** | **dbSNP ID** | **Nucleotide change** | **AA change** | **Molecular consequences** | **1000G MAF** | **1000G MAF (S. Han)** | **GnomAD MAF (Total)** | **GnomAD MAF (East Asian)** | **Taiwan Biobank MAF** | **Allele F in our study** | **p value** |  |
| Chr3: 48,610,058 | rs148974613 | c.-438A>C | - | Upstream variant | G=0.0048 | G=0.0238 | G=0.0009989 | G=0.02290 | G=0.020308 | 0.0140 | 0.796 |  |
| Chr3: 48,609,837 | rs571412356 | c.-217G>A | - | Upstream variant | T=0.0004 | T=0.0000 | T=0.0003087 | T=0.007885 | - | 0.0140 | - |  |
| Chr3: 48,609,535 | rs146649569 | c.69+17A>G | - | Intron variant | C=0.0004 | C=0.0048 | C=0.0001324 | C=0.003662 | C=0.002849 | 0.0047 | 0.485 |  |
| Chr3: 48,609,377 | rs181443725 | c.70-75G>T | - | Intron variant | A=0.0084 | A=0.0000 | A=0.00551 | A=0.0009612 | A=0.001944 | 0.0047 | 0.381 |  |
| Chr3: 48,609,376 | rs191615367 | c.70-74G>T | - | Intron variant | A=0.0084 | A=0.0000 | A=0.005485 | A=0.0009619 | A=0.002451 | 0.0047 | 0.451 |  |
| Chr3: 48,605,869* | rs759847580 | c.211-13G>C | - | Intron variant | - | - | G=0.00001971 | G=0.0005771 | - | 0.0047 | - |  |
| Chr3: 48,605,749 | rs74672788 | c.297+21C>T | - | Intron variant | A=0.0036 | A=0.0095 | A=0.000506 | A=0.0125 | A=0.012525 | 0.0327 | 0.025 |  |
| Chr3: 48,605,724* | rs773170915 | c.297+46T>C | - | Intron variant | - | - | G=0.000006569 | G=0.0001922 | - | 0.0047 | - |  |
| Chr3: 48,604,538 | rs35343040 | c.428-107T>C | - | Intron variant | G=0.1783 | G=0.1238 | G=0.1456 | G=0.1106 | G=0.110561 | 0.1121 | 0.902 |  |
| Chr3: 48,603,627 | rs17080284 | c.643G>C | p.Asp215His  (p.D215H) | Missense variant | G=0.0048 | G=0.0143 | G=0.001196 | G=0.03331 | G=0.036915 | 0.0280 | 0.573 |  |
| Chr3: 48,601,553* | - | c.707-88A>G | - | Intron variant | - | - | - | - | - | 0.0047 | - |  |
| Chr3: 48,601,374 | rs149245457 | c.800C>G | p.Pro267Arg  (p.P267R) | Missense variant | C=0.0110 | C=0.0381 | C=0.001668 | C=0.03985 | C=0.035620 | 0.0327 | 0.852 |  |
| Chr3: 48,601,132* | rs756010041 | c.823-14A>G | - | Intron variant | - | - | C=0.00001972 | C=0.000 | - | 0.0047 | - |  |
| Chr3: 48,601,018 | rs187641562 | c.923A>G | p.Asn308Ser  (p.N308S) | Missense variant | C=0.0004 | C=0.0048 | C=0.0001905 | C=0.003865 | C=0.004944 | 0.0047 | 1.000 |  |
| Chr3: 48,600,961 | rs184725145 | c.966+14C>T | - | Intron variant | A=0.0018 | A=0.0048 | A=0.0002891 | A=0.007523 | A=0.006596 | 0.0047 | 1.000 |  |
| Chr3: 48,600,697* | rs761304717 | c.1110C>T | p.Phe370=  (p.F370=) | Synonymous variant | - | - | A=0.00002629 | A=0.000 | - | 0.0047 | - |  |
| Chr3: 48,600,531 | rs140583334 | c.1164G>T | p.Val388=  (p.V388=) | Synonymous variant | A=0.0072 | A=0.0238 | A=0.001236 | A=0.03080 | A=0.035094 | 0.0421 | 0.554 |  |
| Chr3: 48,599,152 | rs182453765 | c.1419C>T | p.Ser473=  (p.S473=) | Synonymous variant | A=0.0022 | A=0.0048 | A=0.0004206 | A=0.01059 | A=0.011348 | 0.0187 | 0.315 |  |
| Chr3: 48,599,080* | rs575926762 | c.*48 | - | Non coding transcript variant | C=0.0002 | C=0.0000 | C=0.00002628 | C=0.0003848 | - | 0.0047 | - |  |
| Chr3: 48,599,074- 48,599,082* | rs953114128 | c.*54delC | - | Non coding transcript variant | - | - | delG=0.0006752 | delG=0.0005823 | - | 0.0187 | - |  |
| * MAF was <0.001 in all databases (if available) | | | | | | | | | | | |  |
| EOPD, early-onset Parkinson's disease; FPD, familial Parkinson's disease; GRCh38.p12, Genome Reference Consortium Human Build 38 patch release 12; Ref, Reference allele; Alt, Alternative allele; dbSNP, The Single Nucleotide Polymorphism Database; AA, Amino acid; MAF, minor allele frequency; 1000G, 1000 Genomes; S. Han, Southern Han Chinese; gnomAD, The Genome Aggregation Database; F, frequency | | | | | | | | | | | |  |
|  |  |  |  |  |  |  |  |  |  |  |  |  |
| *p* values <0.00417 were considered statistically significant after Bonferroni’s correction was applied. | | | | | | | | | | | |  |

|  | | | | | | | | | | | | | | | | |  |  |  |
| --- | --- | --- | --- | --- | --- | --- | --- | --- | --- | --- | --- | --- | --- | --- | --- | --- | --- | --- | --- |
| **Supplementary Table 4.** In silico pathogenicity predictions for the *UQCRC1* missense variant | | | | | | | | | | | | | | | | | | |  |
| **Genomic location (GRCh38.p12)** | **dbSNP ID** | **Nucleotide change** | **AA change** | **FATHMM** | **Mutation Assessor** | **SIFT** | **SIFT4G** | **Mutation Taster** | **PolyPhen2 HDIV** | **PolyPhen2 HVAR** | **PROVEAN** | **CADD phred** | **M-CAP** | **LRT** | **PrimateAI** | **REVEL** | | |  |
| Chr3: 48,603,627 | rs17080284 | c.643G>C | p.Asp215His  (p.D215H) | T (0.51) | M (2.33) | T (0.097) | T (0.128) | D (1) | D (0.999) | D (0.968) | D (-6.34) | 25.2 | B (-) | D (0) | T (0.6289) | B (0.2569) | | |  |
| Chr3: 48,601,374 | rs149245457 | c.800C>G | p.Pro267Arg  (p.P267R) | T (1.2) | M (2.395) | T (0.051) | D (0.041) | P (0.9959) | B (0.025) | B (0.012) | D (-5.5) | 17.4 | B (-) | D (0.000022) | T (0.3147) | B (0.08699) | | |  |
| Chr3: 48,601,018 | rs187641562 | c.923A>G | p.Asn308Ser  (p.N308S) | T (1.49) | T (1.32) | D (0.258) | T (0.213) | D (1) | B (0.180) | B (0.054) | D (-3.31) | 19.7 | B (0.009) | D (0) | T (0.5025) | B (0.0071) | | |  |
| GRCh38.p12, Genome Reference Consortium Human Build 38 patch release 12; dbSNP, The Single Nucleotide Polymorphism Database; AA, Amino acid; FATHMM, functional analysis through hidden Markov models; SIFT, sorting intolerant from tolerant; PolyPhen2 HDIV, polymorphism phenotyping v2 human diversity; PolyPhen2 HVAR, polymorphism phenotyping v2 human variation; PROVEAN, Protein Variation Effect Analyzer; CADD, combined annotation-dependent depletion; M-CAP, Mendelian clinically applicable pathogenicity; LRT, Likelihood Ratio Test. | | | | | | | | | | | | | | | | | | |  |
|  |  |  |  |  |  |  |  |  |  |  |  |  |  |  |  |  |  |  |  |
|  |  |  |  |  |  |  |  |  |  |  |  |  |  |  |  |  |  |  |  |
| T, tolerated; M, medium; B, benign; D, damaging/probably damaging/disease causing/deleterious; P, polymorphism. | | | | | | | | |  |  |  |  |  |  |  |  | | |  |

| **Supplementary Table 5.** In silico pathogenicity predictions for *UQCRC1* rare noncoding variants | | | | | | | | | |  |
| --- | --- | --- | --- | --- | --- | --- | --- | --- | --- | --- |
| **Genomic location (GRCh38.p12)** | **dbSNP ID** | **Nucleotide change** | **CADD phred** | **MaxEntScan** | **NNSPLICE** | **SpliceAI** | **RegSNP-intron** | **PhyloP100way** | **ACMG pathogenicity classification** |  |
| Chr3: 48,605,869 | rs759847580 | c.211-13G>C | 0.917 | 7.39 (+10.8%) | 0.80 (+19.4%) | 0.01 (Acceptor loss) | B (0.09) | Not strongly conserved (-1.349) | Likely benign |  |
| Chr3: 48,605,724 | rs773170915 | c.297+46T>C | 6.64 | - | - | 0.01 (Donor gain) | B (0.19) | Not strongly conserved (0.901) | Uncertain significance |  |
| Chr3: 48,601,553 | - | c.707-88A>G | 2.232 | - | - | 0.08 (Acceptor gain) | B (0.24) | Not strongly conserved (-0.033) | Uncertain significance |  |
| Chr3: 48,601,132 | rs756010041 | c.823-14A>G | 0.496 | 9.78 (-0.7%) | 0.94 (0%) | 0.09 (Acceptor gain) | B (0.31) | Not strongly conserved (-0.154) | Uncertain significance |  |
| Chr3: 48,599,080 | rs575926762 | c.*48 | 6.80 | - | - | 0.02 (Acceptor loss) | - | Not strongly conserved (0.272) | Likely benign |  |
| Chr3: 48,599,074- 48,599,082 | rs953114128 | c.*54delC | 1.59 | - | - | 0.06 (Acceptor loss) | - | Not strongly conserved (-0.291 to 3.020) | Uncertain significance |  |
| GRCh38.p12, Genome Reference Consortium Human Build 38 patch release 12; dbSNP, The Single Nucleotide Polymorphism Database; CADD, combined annotation-dependent depletion; PhyloP100way, vertebrate Phylogenetic p-values for 100 vertebrate species; ACMG, American College of Medical Genetics and Genomics. | | | | | | | | | |  |
|  |  |  |  |  |  |  |  |  |  |  |
| B, benign. |  |  |  |  |  |  |  |  |  |  |

| **Supplementary Table 6.** In silico pathogenicity predictions for *UQCRC1* rare synonymous variant | | | | | | | | |  |
| --- | --- | --- | --- | --- | --- | --- | --- | --- | --- |
| **Genomic location (GRCh38.p12)** | **dbSNP ID** | **Nucleotide change** | **AA change** | **CADD phred** | **RegSNPs- splicing** | **SpliceAI** | **PhyloP100way** | **ACMG pathogenicity classification** |  |
| Chr3: 48,600,697 | rs761304717 | c.1110C>T | p.Phe370=  (p.F370=) | 9.92 | PoD (0.462) | 0.0100 (acceptor_gain) | Not strongly conserved (1.258) | Likely benign |  |
| GRCh38.p12, Genome Reference Consortium Human Build 38 patch release 12; dbSNP, The Single Nucleotide Polymorphism Database; CADD, combined annotation-dependent depletion; PhyloP100way, vertebrate Phylogenetic p-values for 100 vertebrate species; ACMG, American College of Medical Genetics and Genomics. | | | | | | | | |  |
|  |  |  |  |  |  |  |  |  |  |
|  |  |  |  |  |  |  |  |  |  |
| PoD, potentially deleterious. | |  |  |  |  |  |  |  |  |
